# Seven immune‐related genes prognostic power and correlation with tumor‐infiltrating immune cells in hepatocellular carcinoma

**DOI:** 10.1002/cam4.3406

**Published:** 2020-08-20

**Authors:** Tiantian Liu, Hao Wu, Jianni Qi, Chengyong Qin, Qiang Zhu

**Affiliations:** ^1^ Department of Gastroenterology Shandong Provincial Hospital Cheeloo College of Medicine Shandong University Jinan Shandong China; ^2^ Shandong Provincial Engineering and Technological Research Center for Liver Diseases Prevention and Control Jinan Shandong China; ^3^ Central Laboratory Shandong Provincial Hospital Cheeloo College of Medicine Shandong University Jinan Shandong China; ^4^ Central Laboratory Shandong Provincial Hospital Affiliated to Shandong First Medical University Jinan Shandong China; ^5^ Department of Gastroenterology Shandong Provincial Hospital Affiliated to Shandong First Medical University Jinan Shandong China

**Keywords:** hepatocellular carcinoma, immunology, microenvironment, prognosis

## Abstract

**Background:**

Given poor prognosis and the lack of efficient therapy for advanced hepatocellular carcinoma, immunotherapy has emerged as an increasingly important role. However, there were few reports on the correlation between immune‐related genes and HCC. The purpose of this study is to construct a novel immune‐related gene‐based prognostic signature for HCC and to explore the potential mechanisms.

**Methods:**

We organized expression data of 374 HCC samples and 50 nontumor samples from TCGA database. A robust signature was constructed by Cox regression analysis based on the immune‐related genes, which were filtered by differential genes analysis and Cox regression analysis. Then, the correlation analysis between the signature and clinical characteristics was conducted. And the signature was validated in ICGC database. Furthermore, the relationships between immune cell infiltration and the signature were explored by bioinformatics analysis.

**Results:**

Seven genes‐based model (Risk score = BIRC5 * 0.0238 + FOS * 0.0055 + DKK1 * 0.0085 + FGF13 * 0.3432 + IL11 * 0.0135 + IL17D * 0.0878 + SPP1 * 0.0003) was constructed eventually and it was proved to be an independent prognostic factor for HCC patients. The signature‐calculated risk scores were shown to be positively correlated with the infiltration of these five immune cells, including macrophages, neutrophils, CD8+T, dendritic, and B cells. And the results suggested that high amplication of BIRC5, FGF13, IL11, IL17D, and SPP1 were more likely correlated with immune cell infiltration. Finally, PPI network, TFs‐based regulatory network and gene enrichment plots were performed to show potential molecular mechanisms.

**Conclusion:**

We construct a robust immune‐related gene‐based prognostic signature with seven genes and explore potential mechanisms about it, which may contribute to the immunotherapy research for HCC.

## Graphical abstract

A novel immune‐related gene‐based prognostic signature with 7 genes was constructed. And we also explored the correlationships with it and its potential mechanisms, which may contribute to the immunotherapy research for HCC.

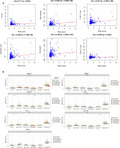

## 1 INTRODUCTION

Liver cancer is one of the most important factors in tumor‐related death, threatening thousands of lives worldwide.^1^, ^2^ The most common pathological type in liver cancer is hepatocellular carcinoma (HCC) which arises from chronic liver inflammation and liver fibrosis mostly.^3^, ^4^ Nowadays, surgical resection and liver transplantation are the most widely used treatments for HCC internationally. However, given that these two treatments show several drawbacks, such as rapid postoperative recurrence and shortage of liver donors, the therapeutic effects are far from satisfactory.^5^ Moreover these two methods have limitations in the treatment of advanced HCC, which is usually diagnosed at late stages, resulting in poor survival rate.^5^ Considering the restrictions and poor outcome of existing treatments, immunotherapy, acting as a treatment strategy, is under intensive investigation currently.^6^, ^7^ Although the immunotherapy of HCC has made some progress, such as medicines of immune checkpoint inhibitors (ICIs),^6^ there are still a lot needed to be explored. Therefore, it is essential to investigate immune‐related genetic profiling of HCC, thereby providing direction for immunotherapy.

Immunotherapy, as one of cancer treatment methods, has been explored for a long time. The development of immunotherapy has attracted more and more attention to tumor immune microenvironment (TIME). Some studies showed that constituents in TIME, including infiltrating immune cells, secreted cytokines/chemokines and other components, were very important in the research of HCC development and the investigation of immunotherapy. The relationships between HCC prognosis and the multiplicity and function of tumor infiltrating immune cells were explored in several studies.^8^, ^9^, ^10^, ^11^ The diversity of immature CD11‐CD27‐NK cells, which are highly expressed in HCC tissue, increases with the progress of liver cancer and has been proved to be a prognostic predictor of HCC patients.^12^ In addition, low CD4+ cytotoxic T cells expression was significantly correlated with prognosis of HCC patients, and was considered to be an independent prognostic predictor for survival time of HCC patients.^10^ The number of neutrophils in HCC infiltrate could also predict poor prognosis for HCC patients after resection.^13^ What's more, several studies have shown that some cytokines in TIME are related to development and progression of HCC.^14^, ^15^, ^16^ Enhanced expression of serum IL6 could increase the risk of HCC development and was associated with early metastasis of HCC.^14^ High expression level of IL10 in plasma could predict short survival time for HCC patients.^16^ According to these evidences, we can conclude that TIME plays an important role in predicting HCC patients’ prognosis. However, there is still a lack of signatures to evaluate TIME based on immune‐related genes and predict HCC patients' prognosis as well. In consequence, it is important to find a powerful signature built on immune‐related genes for HCC which could predict HCC patients’ survival and may provide direction for HCC immunotherapy at the same time.

In this study, an immune‐related gene‐based risk signature was constructed, which could predict prognosis of HCC patients independently. Then, the correlations between the signature and clinical characteristics were evaluated. And the correlations between the signature and immune cell filtration were also explored. Apart from those, potential regulatory mechanisms of this signature were displayed through bioinformatics analysis.

## 2 METHODS

### 2.1 Data resource

RNA‐sequencing data were contained from the TCGA database (The Cancer Genome Atlas database, https://cancergenome.nih.gov/) and the raw count data of 374 HCC samples and 50 nontumor samples were collected. In addition, the full clinical characteristics of corresponding patients were downloaded and extracted. The immune‐related genes were obtained from the ImmPort database (https://immport.niaid.nih.gov) and we got 2498 immune‐related genes.^17^, ^18^ In addition, RNA expression sequencing data and patient clinical information of 232 HCC samples were obtained by ICGC database (https://dcc.icgc.org/) for validation.

### 2.2 Development of the immune‐related gene‐based signature

To get immune genes involved in the onset of HCC, we performed differential expression analysis between HCC and nontumor samples, using the edgeR package in R. All differentially expressed genes were extracted according to *P* value<.05 and the absolute log2 fold change (FC) ≥ 2, and then immune‐related genes were screened from these differentially expressed genes. Univariate Cox regression analysis, basing on differentially expressed immune‐related genes, was conducted to explore the association between expression levels of these genes and HCC patients’ overall survival (OS). Then, genes with statistical significance (*P* < .05) in univariate Cox regression analysis were included in multivariate proportional hazards regression analysis. Differentially expressed genes that were statistically significant in both univariate and multivariate analyses were considered to be independent prognostic factors for HCC and were chosen to develop immune signature. The immune‐related signature was constructed in Cox proportional hazards model method of R package “survival” and then we stratified HCC patients into high‐risk and low‐risk groups based on the median value of risk scores.

### 2.3 Validation of the immune‐related gene‐based signature

In order to verify the predictive performance of signatures, we conducted the Kaplan‐Meier (K‐M) survival analysis through the "survival" package in R to reflect OS of high‐risk and low‐risk groups. And, the ROC curve, which showed the sensitivity and specificity of the signature for predicting prognosis, was demonstrated with R package of “survival ROC.” What's more, expression data from ICGC database was used to conduct external validation. K‐M analysis and ROC curve were performed in the ICGC database by using the R package. To further verify the prognostic capabilities of the signature, we calculated and compared the C‐index in both TCGA and ICGC database.^19^

### 2.4 Relationship between clinical variables and the signature

We performed the correlation analysis the genes that make up the signature and clinicopathologic factor, including tumor grade, pathological stage, age, gender, Alpha fetoprotein (AFP) quantification, and Hepatitis B virus (HBV) infection. In addition, based on the clinical characteristics and the signature as the included variables, we conducted univariate Cox regression analysis and multivariate Cox regression analysis. Then, the figures were made to present the results of the correlation analysis intuitively.

### 2.5 Immune cell infiltration analysis

We conducted an analysis about the relationships between the signature and the amount of six kinds of immune cell infiltration downloaded from TIMER (Tumor Immune Estimation Resource).^20^ Besides, the correlations between copy number variations of the signature constituent genes and the immune cells are explored at TIMER. The TIMER online database offers abundances of tumor‐infiltrating immune cells for multiple tumors, which contains six kinds of cells, including macrophages, neutrophils, B, CD4+T, CD8+T, and dendritic cells.

### 2.6 Regulatory mechanisms analysis

The Protein‐Protein Interaction (PPI) network was displayed based on differentially expressed immune‐related genes that are correlated with HCC patients’ prognosis via the STRING online database (https://string‐db.org/).^21^, ^22^ Then, we performed functional enrichment analyses to explore potential molecular mechanisms of these immune genes via the GO and KEGG pathways in DAVID website (https://david.ncifcrf.gov/).^23^ TFs are vital molecules which regulate the gene expression directly. Therefore, it is essential to explore the potential adjustment ability of TFs to the immune signature genes. The comprehensive list of TFs was collected from Cistrome Cancer database (http://cistrome.org/)^24^ and the regulatory network of genes in the signature and TFs was constructed by Cytoscape.^25^ Gene set enrichment analysis (GSEA) (https://pypi.org/project/gseapy/) was used to analyze the GO term of the genes that make up the signature.^26^

### 2.7 Statistical analysis

R version 3.5.1 was used to conduct the statistical analysis of this research.^18^ And p value < 0.05 was regarded as statistically significant.^18^

## 3 RESULTS

### 3.1 Differential expression analysis

**Figure 1:**
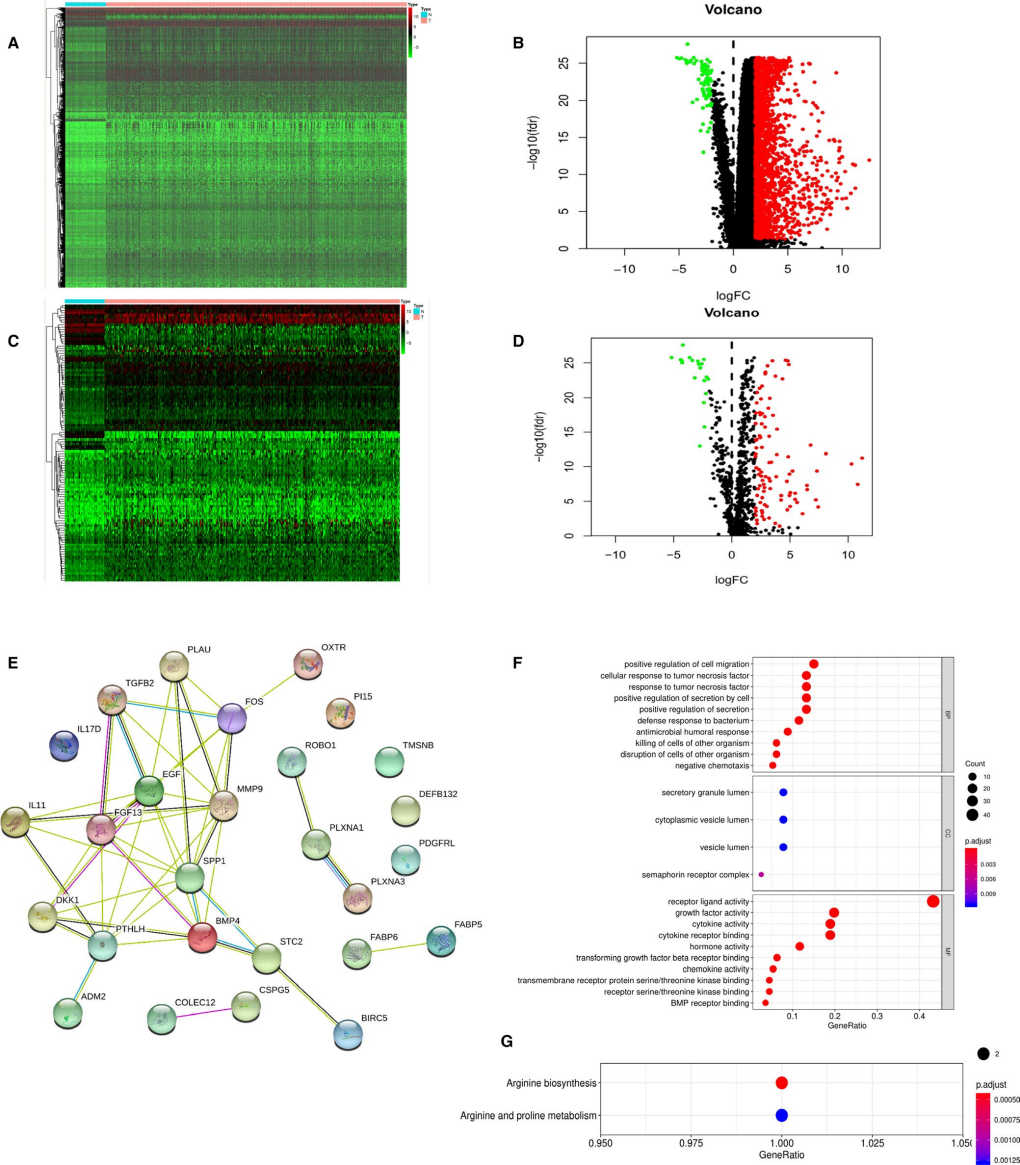
(A) Heatmap of differentially expressed genes in HCC. The color from green to red represents the progression from low expression to high expression. (B) Volcano of differentially expressed genes in HCC. The red dots in the plot represents up‐regulated genes and green dots represents down‐regulated genes with statistical significance. Black dots represent no differentially expressed genes. (C) Heatmap of differentially expressed immune‐related genes in HCC. Red represents higher expression while green represents lower expression. (D) Volcano plot of differentially expressed immune‐related genes in HCC. Colored dots represent differentially expressed immune‐related genes and black dots represent no differentially expressed immune‐related genes. (E) Protein‐protein interaction network of prognostic immune‐related genes. (F) Gene ontology analysis based on prognostic immune‐related genes, including biological process, cellular component and molecular function from top to bottom. (G) The Kyoto Encyclopedia of Genes and Genomes (KEGG) pathway based on prognostic immune‐related genes

We conducted differential expression analysis and a total of 2062 genes (|logFC|>2, *P* < .05) were chosen compared gene expressions of HCC tissues with gene expressions of normal tissues. There were 77 down‐regulated and 198,5 up‐regulated genes among those differentially expressed genes (Figure 1A,B). Then, 116 immune‐related genes, including 20 down‐regulated and 96 up‐regulated genes, were extracted from the differentially expressed genes (Figure 1C, D).

Through the univariate Cox analysis with these 116 genes, a total of 27 genes that were significantly correlated with HCC patients’ survival were extracted, and these genes were considered as factors participating in the occurrence and progression of HCC. To explore the potential mechanisms of these 27 genes, the PPI network based on these 27 genes was constructed using the STRING database, and there were 45 edges and 90 nodes in the PPI network (Figure 1E). According to the numbers of nodes, FGF13, DKK1, FOS, and SPP1 were the possible core genes among the seven immune‐related genes in the PPI network. The GO analysis showed that these 27 prognosis‐associated differentially expressed genes were most enriched in “positive regulation of cell migration” in biological processes, “secretory granule lumen” in cellular components, and “receptor ligand activity” in molecular functions (Figure 1F). And these 27 genes were proved to be actively involved in arginine biosynthesis KEGG according to Functional enrichment analysis (Figure 1G).

### 3.2 Establishment and verification of immune‐related gene‐based signature

Then, the results of univariate and multivariate analysis with the 27 prognosis‐related genes were performed, and nine independent prognostic factors (PTHLH, PLXNA3, BIRC5, FOS, DKK1, FGF13, IL11, IL17D, SPP1) of HCC were extracted according to the results of univariate and multivariate analysis (Table 1). And these nine genes were analyzed by Cox proportional hazards model method of R package “survival” to find the best model. Finally, the seven immune‐related genes were chosen and the signature was constructed as follows:

**TABLE 1:** Univariate and multivariate cox regression analysis of the 27 immune‐related genes

Gene ID	Univariate analysis		Multivariate analysis	
	HR (95%CI)	*P* value	HR (95%CI)	*P* value
TMSB15A	1.04(1.00,1.07)	.030	0.64(0.37,1.14)	.130
DEFB132	0.81(0.66,0.99)	.044	0.82(0.66,1.02)	.075
COLEC12	1.03(1.01,1.06)	.016	1.15(0.97,1.36)	.097
MMP9	1.00(1.00,1.01)	.015	1.00(0.97,1.36)	.740
FABP6	1.07(1.00,1.14)	.045	1.01(0.90,1.13)	.903
PLAU	1.01(1.00,1.01)	.019	1.04(0.95,1.12)	.400
FABP5	1.04(1.01,1.06)	.004	0.98(0.93,1.04)	.514
PI15	1.10(1.01,1.06)	.030	1.01(0.78,1.30)	.968
BIRC5	1.02(1.01,1.04)	.001	1.03(1.00,1.06)	**.039**
FOS	1.00(1.00,1.01)	.040	1.01(1.00,1.01)	**.019**
PLXNA1	1.15(1.08,1.23)	<.001	1.11(0.97,1.26))	.136
PLXNA3	1.15(1.03,1.29)	.015	0.71(0.56,0.91)	**.006**
ROBO1	1.02(1.00,1.03)	.031	1.00(0.98,1.03)	.735
ADM2	1.04(1.00,1.03)	.032	1.04(0.99,1.09)	.084
BMP4	1.02(1.00,1.03)	.015	1.00(0.97,1.02)	.805
CSPG5	1.34(1.02,1.76)	.037	1.31(0.91,1.89)	.151
DKK1	1.01(1.00,1.01)	<.001	1.01(1.00,1.01)	**<.001**
EGF	1.40(1.11,1.76)	.004	1.07(0.76,1.50)	.691
FGF13	1.31(1.05,1.63)	.017	1.43(1.05,1.95)	**.024**
IL11	1.02(1.00,1.03)	.015	1.05(1.00,1.09)	**.035**
IL17D	1.08(1.02,1.15)	.007	1.10(1.04,1.18)	**<.001**
PDGFRL	1.10(1.01,1.18)	.022	1.00(0.89,1.12)	.990
PTHLH	1.01(1.00,1.02)	.018	1.02(1.00,1.03)	**.020**
SPP1	1.00(1.00,1.00)	<.001	1.00(1.00,1.03)	**.009**
STC2	1.03(1.01,1.05)	.003	1.01(0.98,1.05)	.382
TGFB2	1.05(1.02,1.07)	.001	1.06(0.95,1.18)	.299
OXTR	1.04(1.01,1.06	.005	0.98(0.91,1.06)	.604

Note

Bold numbers indicate significance at ≤ .05

Riskscore=BIRC5*0.0238+FOS*0.0055+DKK1*0.0085+FGF13*0.3432+IL11*0.0135+IL17D*0.0878+SPP1*0.0003.

**Figure 2:**
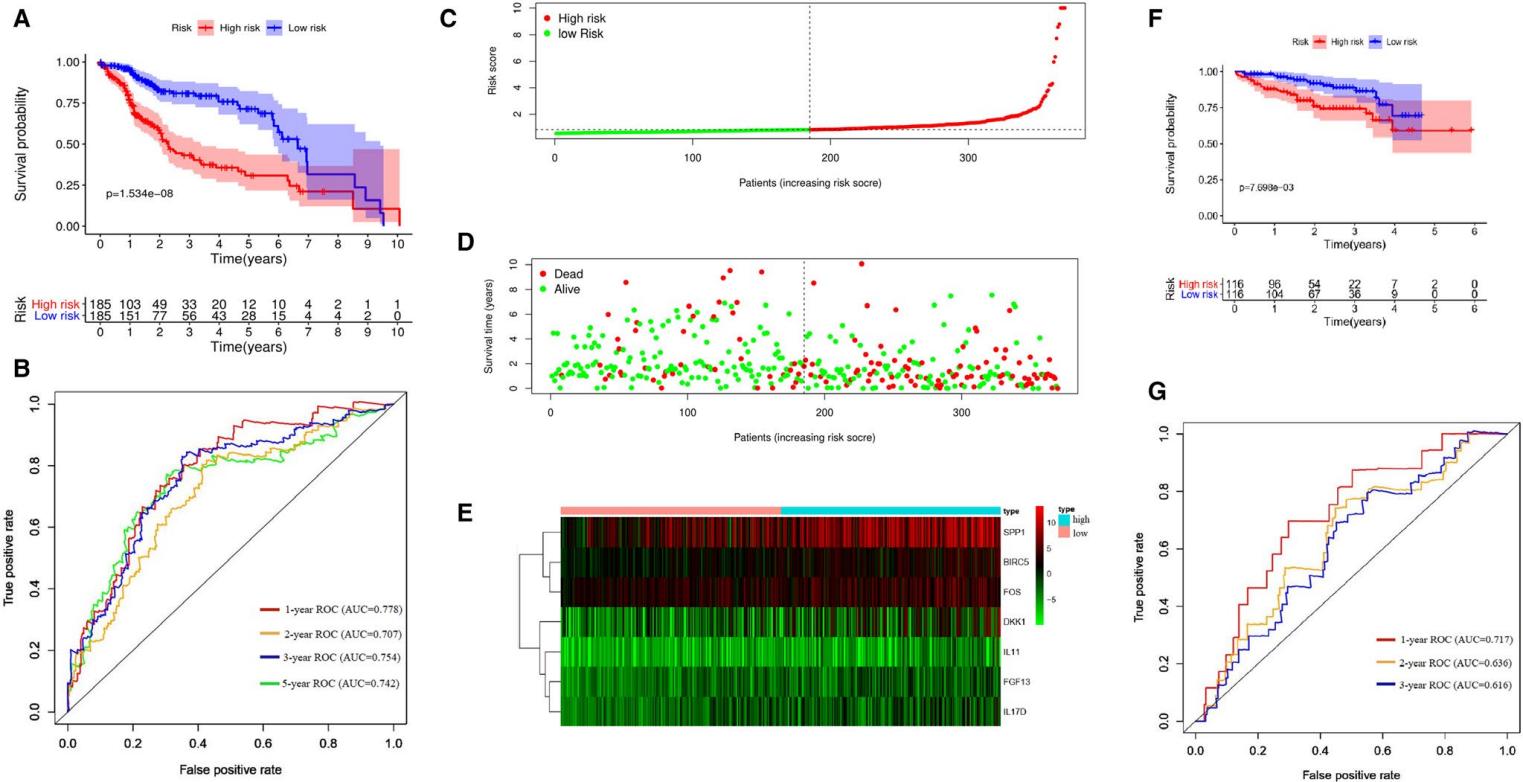
(A) Survival curve for the high risk and low risk groups of HCC patients in the TCGA database. (B) Receiver operating characteristic (ROC) curve of the prognostic signature in TCGA database. (C) The risk score distribution of HCC patients in the TCGA database. (D) Survival status of patients in high‐risk and low‐risk groups. (E) Heatmap of the seven immune‐related genes expression in HCC patients. (F) Survival curve for the high risk and low risk groups of HCC patients for validation in ICGC database. (G) Receiver operating characteristic (ROC) curve of the prognostic signature for validation in ICGC database

What's more, taking the median risk score as the cut‐off value, HCC patients were grouped into high‐risk and low‐risk groups. According to the result of K‐M analysis, the higher the risk score calculated by the signature of HCC patients, the worse the prognosis (*P* < .001, Figure 2A). The 1‐year, 3‐year, and 5‐year ROC curve analysis showed that the area under the curve (AUC) was 0.778, 0.754, and 0.742, respectively (Figure 2B). These results showed that the signature we constructed has a good predictive effect on the survival of HCC patients. Then, we also depicted the distributions of risk score and survival status, which suggested that there were more deaths in high‐risk group (Figure 2C, D). What's more, heatmap showed the distribution of the expression of seven immune‐related genes. Compared with the low‐risk group, all the seven genes showed relatively high expression in the high‐risk group (Figure 2E).

To verify the predictive power of the immune‐related genes‐based signature furtherly, 232 HCC patients from ICGC database were used for external validation. And we divided the patients into high risk (n = 116) and low risk groups (n = 116) at cutoff value of median risk score. Consistent with the result in TCGA dataset, K‐M analysis showed that patients in the high‐risk group had a worse prognosis than those in the low‐risk group (*P* = .007698, Figure 2F). Then, ROC curves were performed and the results showed that the 1‐year AUC, 2‐year AUC, and 3‐year AUC was 0.717, 0.636, and 0.616, respectively (Figure 2G). There are only two patients with OS longer than 5 years, so the AUC of 5‐year ROC curve is not calculated. The C‐index was 0.699 and 0.754 in TCGA dataset and ICGC dataset, respectively, suggesting the strong predictive power of the signature.

### 3.3 Relationship of the immune‐related gene‐based signature with clinical variables

**Figure 3:**
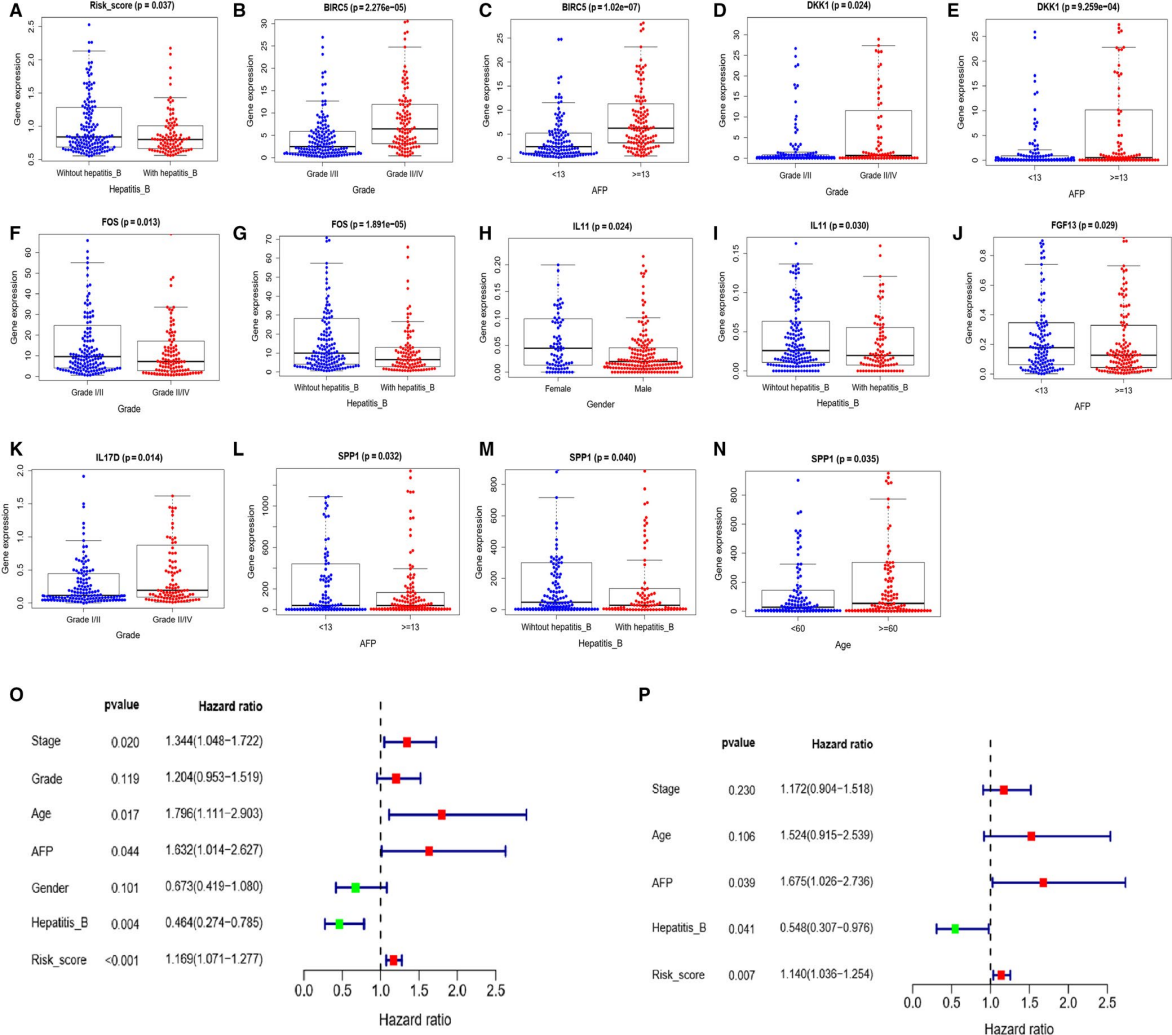
Relationships between risk score, expression of the seven immune‐related genes and clinical characteristics. (A) Relationship between risk score and hepatitis B. (B) Relationship between BIRC5 and grade. (C) Relationship between BIRC5 and AFP. (D) Relationship between DKK1 and grade. (E) Relationship between DKK1 and AFP. (F) Relationship between FOS and grade. (G) Relationship between FOS and hepatitis B. (H) Relationship betweenIL11 and gender. (I) Relationship between IL11 and hepatitis B. (J) Relationship between FGF13 and hepatitis B (K) Relationship between IL17D and grade. (L) Relationship between FOS and AFP. (M) Relationship between FOS and hepatitis B. (N) Relationship between FOS and age. (O) Forest plot for univariate cox analysis. (P) Forest plot for multivariate cox analysis

Afterward, we explored the correlations between clinicopathologic factors and risk scores calculated from the immune‐related gene‐based signature. The correlations between the seven immune‐related genes and clinical characteristics were also assessed. The figures and the table demonstrated that HBV tended to correlated with lower risk scores (*P* = .037, Figure 3A, Table 2). As Figure 3 showed, higher expression of BIRC5 and DKK1 were related with higher level of AFP and higher level of grade (Figure 3B,C,D,E). Higher expression of FOS was related with non‐HBV and lower level of grade (Figure 3F,G). Higher expression of IL11 was related with non‐HBV and females (Figure 3H,I). Higher expression of FGF13 was relevant to lower level of AFP (Figure 3J). And lower expression of IL17D was related with high level of grade (Figure 3K). The low expression of SPP1 is closely related to the high expression of AFP, young people, and HBV infection (Figure 3L,M,N).

**TABLE 2:** Correlations between the seven immune‐related genes and clinical characteristics

Gene ID	Stage	Grade	Age	AFP	Gender	Hepatitis B
BIRC5	−1.339 (0.186)	−**4.333 (<0.001)**	−0.229 (0.819)	−**5.517 (<0.001)**	0.951 (0.343)	−0.296 (0.767)
FOS	−0.217 (0.829)	**2.516 (0.013)**	0.221 (0.825)	0.298 (0.766)	−0.163 (0.871)	**4.377 (<0.001)**
DKK1	−0.976 (0.333)	−**2.274 (0.024)**	0.454 (0.650)	−**3.386 (<0.001)**	1.098 (0.275)	0.805 (0.421)
FGF13	−0.084 (0.933)	0.481 (0.631)	−0.925 (0.356)	**2.210 (0.029)**	−0.232 (0.816)	1.170 (0.243)
IL11	−0.778 (0.439)	−1.629 (0.105)	−0.021 (0.983)	−1.198 (0.232)	**2.287 (0.024)**	**2.181 (0.030)**
IL17D	1.951 (0.053)	−**2.481 (0.014)**	−0.32 (0.749)	−1.170 (0.243)	1.650 (0.102)	−0.679 (0.498)
SPP1	−0.123 (0.903)	−0.693 (0.489)	−**2.125 (0.035)**	**2.162 (0.032)**	−1.634 (0.104)	**2.064 (0.040)**
Risk score	−0.932 (0.355)	−1.585 (0.115)	0.153 (0.878)	−1.44 (0.152)	0.867 (0.388)	**2.096 (0.037)**

Bold numbers indicate significance at ≤ 0.05.

Abbreviations: AFP, alpha fetoprotein; Hepatitis B, Hepatitis B virus.

To testify whether the signature could serve as an independent prognostic factor for OS, univariate and multivariate Cox regression analysis were performed with the signature and clinical characteristics including AFP, stage, grade, age, gender, and HBV infection. In univariate analysis, we could see that risk scores calculated from the signature (HR 1.169, 95%CI 1.071‐1.277, *P* < .001), stage (HR 1.344, 95%CI 1.048‐1.722, *P* = .020), age (HR 1.796, 95%CI 1.111‐2.903, *P* = .017), HBV infection (HR 0.464, 95%CI 0.274‐0.785, *P* = .004), and AFP (HR 1.632, 95%CI 1.014‐2.627, *P* = .044) were connected with OS of HCC patients(Figure 3O). The factors with significant differences in univariate analysis were included in multivariate analysis, and the result suggested that the signature (HR 1.140, 95%CI 1.036‐1.254, *P* = .007), AFP (HR 1.675, 95%CI 1.026‐2.736, *P* = .039), and hepatitis B (HR 0.548, 95%CI 0.307‐0.976, *P* = .041) could predict HCC patients’ OS independently (Figure 3P).

### 3.4 Association between immune cell infiltration and immune‐related gene‐based signature

**Figure 4:**
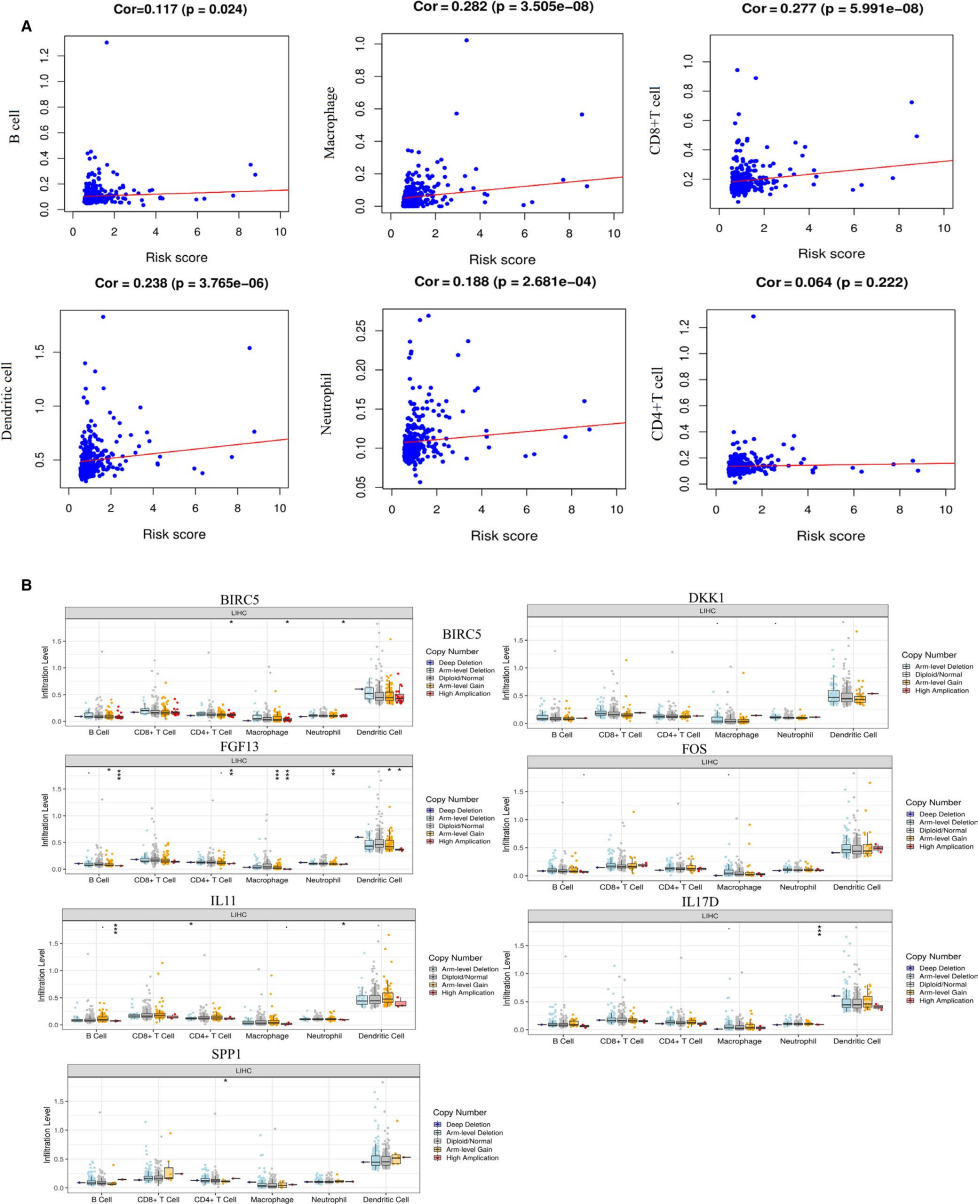
(A) Relationships between the risk score of the signature and 6 types immune cells infiltration. (B) Relationships between copy number variations of the seven immune‐related genes and 6 types immune cells. * represents *P* < .05, ** represents *P* < .01, ***represents *P* < .001

To evaluate the degree of the signature reflecting tumor immune microenvironment, the relationship between the signature and tumor‐infiltrating immune cells were analyzed (Figure 4A). The results revealed that 5 kinds of immune cells including neutrophil (Cor = 0.188, *P* < .001), CD8 + T cell (Cor = 0.277, *P* < .001), dendritic cell(Cor = 0.238, *P* < .001), B cell (Cor = 0.117, *P* = .024), and macrophage (Cor = 0.282, *P* < .001), were positively correlated with the signature significantly. According to Figure 4B, copy number variation analysis showed that high amplication of BIRC5 was associated with CD4 + T cell (*P* < .05), macrophage (*P* < .05) and neutrophil (*P* < .05). Besides, high amplication of FGF13 was correlated with B cell (*P* < .001), CD4 + T cell (*P* < .01), macrophage (*P* < .001) and dendritic cell (*P* < .05) and arm level gain of FGF13 was related to B cell (*P* < .05), macrophage (*P* < .001), neutrophil (*P* < .01) and dendritic cell (*P* < .05). High amplication of IL11 was correlated with neutrophil (*P* < .05) and B cell (*P* < .001), while arm level deletion of IL11 was associated with CD4 + T cell (*P* < .05). And high amplication of IL17D and SPP1 related to neutrophil (*P* < .001) and CD4 + T cell (*P* < .05), respectively.

### 3.5 Mechanisms of the immune‐related signature

**Figure 5:**
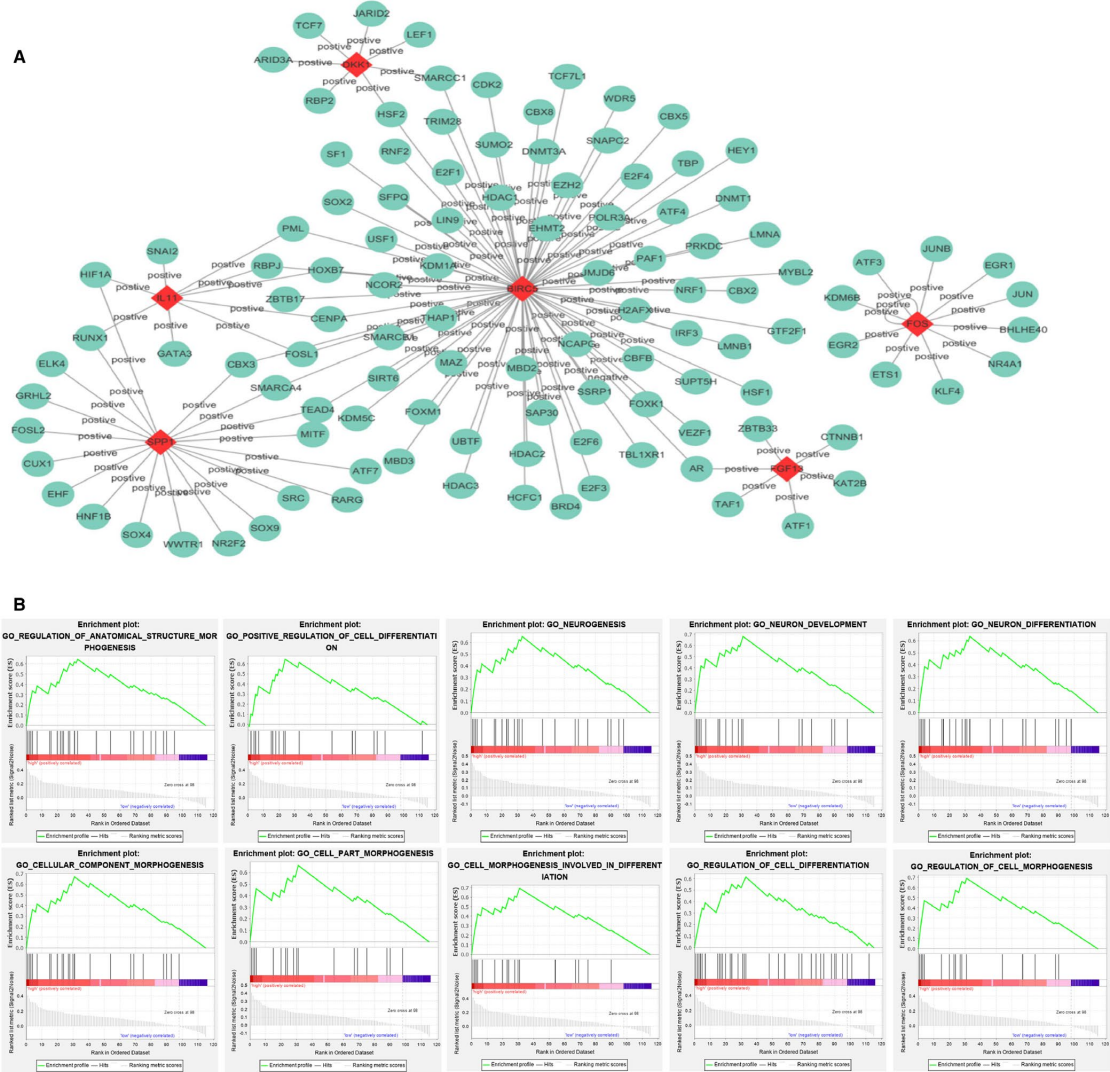
Mechanisms of the immune‐related risk signature. (A) Transcription factors‐based regulatory network with the seven immune‐related genes in the signature. (B) The top 10 significant enrichment GO terms in high‐risk group

We explored the potential regulatory mechanisms of the seven immune‐related genes that made up the signature, which could reflect the regulatory mechanisms of the signature. Based on 315 transcription factors (TF) and seven immune‐related genes, we constructed a relevant regulatory network with correlation coefficient greater than 0.3 as cut‐off value. The regulatory relationships between these TFs and immune‐related genes were revealed in the regulatory network (Figure 5A). In terms of the figure, we can see that BIRC5, FOS, DKK1, FGF13, IL11, and SPP1 were included in the regulatory network, while there was no regulatory relationship between the TFs and IL17D.

The functional enrichment analysis of the immune‐related signature was conducted by GSEA. The top 10 GO terms enriched in high‐risk group were demonstrated in Figure 5B, including cellular component morphogenesis, positive regulation of cell differentiation, regulation of anatomical structure morphogenesis, neuron development, neurogenesis, cell morphogenesis involved in differentiation, neuron differentiation, cell part morphogenesis, regulation of cell differentiation and regulation of cell morphogenesis.

## 4 DISCUSSION

HCC is one of the most common cancers worldwide, and the incidence and mortality of HCC have continued to increase.^1^ In the last several decades, the treatment of HCC has experienced great improvements, including the breakthroughs of immunotherapy.^6^, ^27^, ^28^, ^29^ Considering the importance of the immune environment in the development of tumors and the close connection between immune environment and immunotherapy,^30^ it is crucial to find immune‐related biomarkers that can predict the prognosis of HCC patients, which can also provide guidance for immunotherapy research. In this investigation, we constructed a novel signature on the basis of immune‐related genes for HCC patients, which may also predict HCC patients’ OS independently. Most importantly, we also explored the molecular mechanisms related with the signature using the bioinformatic system and correlations between our signature and immune cell infiltrations to assess immune cells infiltration. These findings indicate that our signature is of great significance for the study of predicting the prognosis of HCC patients and for the exploration of potential immunotherapy targets of HCC.

The signature was constructed by seven immune‐related genes that could predict prognosis of HCC patients. In our study, all the seven immune‐related genes were proved to be independent prognostic markers for HCC patients. Researches indicated that BIRC5 is overexpressed in various malignant tumors, and high expression level of BIRC5 predicted the poor clinical prognosis of these malignant tumor patients, such as malignant breast cancer,^31^ lung adenocarcinoma,^32^ neuroblastic malignant tumor,^33^ and malignant esophageal cancer.^34^ The view consistent with our findings is that BIRC5 is highly expressed in HCC tissue and can predict the prognosis of HCC patients, indicating that BIRC5 could regulate the occurrence and development of HCC.^35^ What's more, Yu Bin et al suggested that DKK1 was overexpressed in HCC tissues for the first time and they demonstrated that DKK1 overexpression is closely related to nuclear/cytoplasmic β‐catenin accumulation in HCC tissues.^36^, ^37^ Studies have shown that the absence of specific c‐Fos in HCC cells can prevent dEn‐induced HCC, while liver‐specific c‐Fos Expression can lead to cell carcinogenesis and enhanced carcinogenesis of dEn.^38^ Meanwhile, it has been reported that IL‐11 could promote the progression of colorectal cancer, breast cancer and gastric cancer.^39^ It was also showed that IL11could participate in the regulation pathways in HCC recurrence and metastasis.^40^ Then, Sun Xiangjun et al demonstrated that the DNA hypomethylation status of IL17D is significantly related to the poor survival of HCC.^41^ In addition, SPP1 could mediate macrophage polarization and immune escape of A549 cells by up‐regulating apoptotic ligand 1 (PD‐L1). In addition, SPP1 may also affect the immune escape and malignant biological behavior of tumor cells by regulating EGFR activation.^42^ Overexpression of SPP1 promotes the development and metastasis of HCC effectively, and may become a underlying target for the treatment of metastatic HCC.^43^, ^44^ SPP1 is a key factor that mediates the polarization process of macrophages, and could provide new ideas for tumor immunotherapy strategies.^45^ At present, there is no research on FGF13 in HCC. From previous studies, exploring the function and mechanism of these seven genes could facilitate the development of new strategies for HCC treatment.

In our study, we systematically evaluated the correlation between the signature and clinicopathological factors. The results showed that patients with HBV infection tended to get high immune risk score. And it can be further confirmed that the immune‐related risk signature, AFP and HBV are independent predictors for prognosis of HCC patients. In addition, the significant relationship between the signature and OS can also suggest that the differences in immune infiltration of HCC may reflect HCC patient's OS. Therefore, it could be a promising predictor for OS of HCC patients by combining the signature and other factors in the future.

Furthermore, the underlying molecular mechanisms of the immune‐related gene‐based signature were also investigated. The TF‐based regulatory network was constructed to guide mechanism analysis of immunotherapy in the future. Among these TFs, HIF1A induces the up‐regulation of PD‐L1 under hypoxic conditions and it could bind to the transcriptionally active hypoxia response element (HRE) in the PD‐L1 proximal promoter, which means that some TFs could play key role in tumor immunotherapy.^46^ The TIME is an important aspect of the study of cancer biology and play a vital role for the occurrence, development and treatment responses of tumors.^47^, ^48^ The components of the immune system, including cells and molecules, are the basic components of TIME. Therefore, it is necessary to illustrate the relationships between HCC immune cell infiltration and the signature to display the status of HCC TIME. According to our results, high‐risk patients tended to related with higher content of B cells, CD8+T cells, dendritic cells, neutrophils and macrophages. In HCC patients, the higher the risk score calculated according to the signature, the higher the amount of immune cell infiltration. And the expression and role of immune cells in HCC have also been reported in previous studies. Zheng C et al revealed the detailed characteristics of infiltrating T cells in HCC from multiple aspects, including clustering, dynamics and developmental trajectory, and the results showed that Tregs and exhausted CD8+T cells are most abundantly expressed in HCC.^49^ Liver tumor infiltration lymphocytes was dominated by CD8+T cells in the tumor tissues and CD4+T cells were higher than CD8+T cells in peritumoral area.^50^ Shalapour S demonstrated that suppression of CD8+T cells played a vital role in driving oncogenesis through IgA‐producing cells for patients with HCC.^51^ In addition, the high expression of tumor infiltrating T cell genes is associated with a better prognosis of anti‐PD‐1 monotherapy.^52^ However, the effects and roles of immune cells in HCC have not been fully explained. Furthermore, we also explored the relationships between copy number variations of the seven immune‐related genes (BIRC5, FOS, DKK1, FGF13, IL11, IL17D and SPP1) and immune cell infiltrations. And it suggested that high amplication of BIRC5, FGF13, IL11, IL17D and SPP1 were more likely correlated with immune cell infiltration. Our preliminary results could provide an orientation to explore the relationships between immune cells and HCC and thorough studies are still necessary.

Recently, we have seen successful clinical results which have promoted many relevant studies and clinical trials, including HCC immunotherapy studies. Immunotherapies targeting programmed death‐1 (PD‐1) and cytotoxic T‐lymphocyte‐associated antigen 4 (CTLA‐4) are becoming effective novel treatment strategies for HCC.^28^, ^29^, ^53^ A recent phase II trial has suggested that pembrolizumab (anti­PD­1) could be a treatment option for advanced HCC.^29^ In addition, some studies have revealed that tremelimumab alone and in combination with ablation show positive effect on HCC treatments.^27^, ^53^ These findings suggest an important role for TIME and the efficacy of immunotherapy for HCC. Some studies have shown that immune‐related elements, such as tumor‐associated macrophages, dendritic cells and B cells, have impact on survival outcomes of patients with HCC.^54^, ^55^, ^56^ Xiao gang et al suggested that up‐regulated FOS promotes the expression of PD‐1 and the disruption of binding of FOS to the AP‐1‐binding site in the gene encoding PD‐1 promoter could enhance antitumor function of T cell.^57^ In addition, chronic lymphocytic leukemia (CLL) cells with down‐regulation of CTLA4 exhibit FOS phosphorylation, and the microenvironment‐controlled CTLA4 expression mediates the proliferation/survival of CLL cells by regulating the expression/activation of FOS.^58^ As a result, with the progress of HCC immunotherapy, the signature and the relationships between the signature and immune cells we have explored could provide further research ideas.

In summary, our study constructed a seven immune‐related gene‐based risk signature and it could predict the prognosis of HCC patients independently. And the signature could reflect several aspects of the HCC microenvironment, which provides potential directions for research on HCC immunotherapy. Nevertheless, the immune‐related genes‐based signature needs to be applied in clinical patients to further verify its prognostic predictability.

## CONFLICTS OF INTEREST

The authors declare that they have no conflict of interest.

## AUTHOR CONTRIBUTION

TL and HW contributed to the design of the study protocol and collected data. CQ, TL, and JQ performed statistical analysis. TL, HW, and QZ performed the figures. TL, CQ, JQ, and QZ contributed to the writing of the manuscript. All authors approved the final version of manuscript.

## ETHICAL APPROVAL

All procedures performed in studies involving human participants were in accordance with the ethical standards of the institutional and/or national research committee and with the 1964 Helsinki declaration and its later amendments or comparable ethical standards. For this type of study formal consent is not required.

## Data Availability

The data that support the findings of this study are available in The Cancer Genome Atlas database at https://cancergenome.nih.gov/, ImmPort database at https://immport.niaid.nih.gov and International Cancer Genome Consortium database at https://dcc.icgc.org.

## References

[1] Bray FI , Ferlay J , Soerjomataram I , Siegel RL , Torre LA , Jemal A . Global cancer statistics 2018: GLOBOCAN estimates of incidence and mortality worldwide for 36 cancers in 185 countries. CA Cancer J Clin 2018;68(6):394‐424. 30207593 10.3322/caac.21492

[2] Nguyen K , Jack K , Sun W . Hepatocellular carcinoma: past and future of molecular target therapy. Diseases (Basel, Switzerland). 2015;4(1). 10.3390/diseases4010001 PMC5456309 28933381

[3] Uehara T , Ainslie GR , Kutanzi K , et al. Molecular mechanisms of fibrosis‐associated promotion of liver carcinogenesis. Toxicol Sci. 2013;132(1):53‐63. 23288052 10.1093/toxsci/kfs342 PMC3576012

[4] Dapito D , Mencin A , Gwak G‐Y , et al. Promotion of hepatocellular carcinoma by the intestinal microbiota and TLR4. Cancer Cell. 2012;21(4):504‐516. 22516259 10.1016/j.ccr.2012.02.007 PMC3332000

[5] Ally A , Balasundaram M , Carlsen R , et al. Comprehensive and integrative genomic characterization of hepatocellular carcinoma. Cell. 2017;169(7):1327‐1341. 28622513 10.1016/j.cell.2017.05.046 PMC5680778

[6] Reig M , Fonseca LGD , Faivre S . New trials and results in systemic treatment of HCC. J Hepatol. 2018;69(2):525‐533. 29653122 10.1016/j.jhep.2018.03.028

[7] Cariani E , Missale G . Immune landscape of hepatocellular carcinoma microenvironment: Implications for prognosis and therapeutic applications. Liver International. 2019;39(9):1608‐1621. 31314948 10.1111/liv.14192

[8] Taketomi MDA , Shimada M , Shirabe MDK , Kajiyama MDK , Gion MDT , Sugimachi MDK . Natural killer cell activity in patients with hepatocellular carcinoma: A new prognostic indicator after hepatectomy. Cancer. 1998;83(1):58‐63. 9655293 10.1002/(sici)1097-0142(19980701)83:1<58::aid-cncr8>3.0.co;2-a

[9] Hoechst B , Voigtlaender T , Ormandy LA , et al. Myeloid derived suppressor cells inhibit natural killer cells in patients with hepatocellular carcinoma via the NKp30 receptor. Hepatology (Baltimore, MD). 2009;50(3):799‐807. 10.1002/hep.23054 PMC6357774 19551844

[10] Fu J , Zhang Z , Zhou L , et al. Impairment of CD4+ cytotoxic T cells predicts poor survival and high recurrence rates in patients with hepatocellular carcinoma. Hepatology (Baltimore, MD). 2013;58(1):139‐149. 10.1002/hep.26054 22961630

[11] Flecken T , Schmidt N , Hild S , et al. Immunodominance and functional alterations of tumor‐associated antigen‐specific CD8+ T‐cell responses in hepatocellular carcinoma. Hepatology (Baltimore, MD). 2014;59(4):1415‐1426. 10.1002/hep.26731 PMC4139003 24002931

[12] Zhang QF , Yin WW , Xia Y , et al. Liver‐infiltrating CD11b(‐)CD27(‐) NK subsets account for NK‐cell dysfunction in patients with hepatocellular carcinoma and are associated with tumor progression. Cell Mol Immunol. 2017;14(10):819‐829. 27321064 10.1038/cmi.2016.28 PMC5649104

[13] Zhou SL , Zhou ZJ , Hu ZQ , et al. Tumor‐associated neutrophils recruit macrophages and t‐regulatory cells to promote progression of hepatocellular carcinoma and resistance to sorafenib. Gastroenterology. 2016;150(7):1646‐1658.e1617. 26924089 10.1053/j.gastro.2016.02.040

[14] Zhang M , Zhang S , Yang Z , et al. Association between the expression levels of IL‐6 and IL‐6R in the hepatocellular carcinoma microenvironment and postoperative recurrence. Oncol Lett. 2018;16(6):7158‐7165. 30546452 10.3892/ol.2018.9557 PMC6256737

[15] Okumoto K , Hattori E , Tamura K , et al. Possible contribution of circulating transforming growth factor‐beta1 to immunity and prognosis in unresectable hepatocellular carcinoma. Liver Int. 2004;24(1):21‐28. 15101997 10.1111/j.1478-3231.2004.00882.x

[16] Chau GY , Wu CW , Lui WY , et al. Serum interleukin‐10 but not interleukin‐6 is related to clinical outcome in patients with resectable hepatocellular carcinoma. Ann Surg. 2000;231(4):552‐558. 10749617 10.1097/00000658-200004000-00015 PMC1421032

[17] Bhattacharya S , Andorf S , Gomes L , et al. ImmPort: disseminating data to the public for the future of immunology. Immunol Res. 2014;58(2–3):234‐239. 24791905 10.1007/s12026-014-8516-1

[18] Wang Z , Song Q , Yang Z , Chen J , Shang J , Ju W . Construction of immune‐related risk signature for renal papillary cell carcinoma. Cancer Med. 2019;8(1):289‐304. 30516029 10.1002/cam4.1905 PMC6346237

[19] Gao X , Yang J , Chen Y . Identification of a four immune‐related genes signature based on an immunogenomic landscape analysis of clear cell renal cell carcinoma. J Cell Physiol. 2020. 10.1002/jcp.29796 32452055

[20] Li T , Fan J , Wang B , et al. TIMER: A web server for comprehensive analysis of tumor‐infiltrating immune cells. Can Res. 2017;77(21):e108‐e110. 10.1158/0008-5472.CAN-17-0307 PMC6042652 29092952

[21] von Mering C , Huynen M , Jaeggi D , Schmidt S , Bork P , Snel B . STRING: a database of predicted functional associations between proteins. Nucleic Acids Res. 2003;31(1):258‐261. 12519996 10.1093/nar/gkg034 PMC165481

[22] Lin P , Guo YN , Shi L , et al. Development of a prognostic index based on an immunogenomic landscape analysis of papillary thyroid cancer. Aging. 2019;11(2):480‐500. 30661062 10.18632/aging.101754 PMC6366981

[23] Dennis G Jr , Sherman BT , Hosack DA , et al. DAVID: database for annotation, visualization, and integrated discovery. Genome Biol. 2003;4(5):P3.12734009

[24] Mei S , Meyer CA , Zheng R , et al. Cistrome cancer: A web resource for integrative gene regulation modeling in cancer. Can Res. 2017;77(21):e19‐e22. 10.1158/0008-5472.CAN-17-0327 PMC5826647 29092931

[25] Shannon P , Markiel A , Ozier O , et al. Cytoscape: a software environment for integrated models of biomolecular interaction networks. Genome Res. 2003;13(11):2498‐2504. 14597658 10.1101/gr.1239303 PMC403769

[26] Subramanian A , Tamayo P , Mootha VK , et al. Gene set enrichment analysis: a knowledge‐based approach for interpreting genome‐wide expression profiles. Proc Natl Acad Sci USA. 2005;102(43):15545‐15550. 16199517 10.1073/pnas.0506580102 PMC1239896

[27] Duffy AG , Ulahannan SV , Makorova‐Rusher O , et al. Tremelimumab in combination with ablation in patients with advanced hepatocellular carcinoma. J Hepatol. 2017;66(3):545‐551. 27816492 10.1016/j.jhep.2016.10.029 PMC5316490

[28] El‐Khoueiry AB , Sangro B , Yau T , et al. Nivolumab in patients with advanced hepatocellular carcinoma (CheckMate 040): an open‐label, non‐comparative, phase 1/2 dose escalation and expansion trial. The Lancet. 2017;389(10088):2492‐2502. 10.1016/S0140-6736(17)31046-2 PMC7539326 28434648

[29] Zhu AX , Finn RS , Edeline J , et al. Pembrolizumab in patients with advanced hepatocellular carcinoma previously treated with sorafenib (KEYNOTE‐224): a non‐randomised, open‐label phase 2 trial. Lancet Oncol. 2018;19(7):940‐952. 29875066 10.1016/S1470-2045(18)30351-6

[30] Chen DS , Mellman I . Elements of cancer immunity and the cancer‐immune set point. Nature. 2017;541(7637):321‐330. 28102259 10.1038/nature21349

[31] Hamy AS , Bieche I , Lehmann‐Che J , et al. BIRC5 (survivin): a pejorative prognostic marker in stage II/III breast cancer with no response to neoadjuvant chemotherapy. Breast Cancer Res Treat. 2016;159(3):499‐511. 27592112 10.1007/s10549-016-3961-2

[32] Cao Y , Zhu W , Chen W , Wu J , Hou G , Li Y . Prognostic value of BIRC5 in lung adenocarcinoma lacking EGFR, KRAS, and ALK mutations by integrated bioinformatics analysis. Dis Markers. 2019;2019:5451290. 31093306 10.1155/2019/5451290 PMC6481100

[33] Hagenbuchner J , Kiechl‐Kohlendorfer U , Obexer P , Ausserlechner M . BIRC5/Survivin as a target for glycolysis inhibition in high‐stage neuroblastoma. Oncogene. 2016;35(16):2052‐2061. 26148234 10.1038/onc.2015.264

[34] Shang X , Liu G , Zhang Y , et al. Downregulation of BIRC5 inhibits the migration and invasion of esophageal cancer cells by interacting with the PI3K/Akt signaling pathway. Oncology Letters. 2018;16(3):3373‐3379. 30127937 10.3892/ol.2018.8986 PMC6096085

[35] Tian Q , Wu Y , Liu Y , et al. Expressions and correlation analysis of HIF‐1alpha, survivin and VEGF in patients with hepatocarcinoma. Eur Rev Med Pharmacol Sci. 2018;22(11):3378‐3385. 29917189 10.26355/eurrev_201806_15159

[36] Yu B , Yang X , Xu Y , et al. Elevated expression of DKK1 is associated with cytoplasmic/nuclear β‐catenin accumulation and poor prognosis in hepatocellular carcinomas. J Hepatol. 2009;50(5):948‐957. 19303159 10.1016/j.jhep.2008.11.020

[37] Chen C , Zhou H , Zhang X , Ma X , Liu Z , Liu X . Elevated levels of Dickkopf‐1 are associated with β‐catenin accumulation and poor prognosis in patients with chondrosarcoma. PLoS One. 2014;9(8):e105414. 25144498 10.1371/journal.pone.0105414 PMC4140757

[38] Bakiri L , Hamacher R , Graña O , et al. Liver carcinogenesis by FOS‐dependent inflammation and cholesterol dysregulation. J Exp Med. 2017;214(5):1387‐1409. 28356389 10.1084/jem.20160935 PMC5413325

[39] Putoczki TL , Ernst M . IL‐11 signaling as a therapeutic target for cancer. Immunotherapy. 2015;7(4):441‐453. 25917632 10.2217/imt.15.17

[40] Wang D , Zheng X , Fu B , et al. Hepatectomy promotes recurrence of liver cancer by enhancing IL‐11‐STAT3 signaling. EBioMedicine. 2019;46:119‐132. 31375423 10.1016/j.ebiom.2019.07.058 PMC6711863

[41] Sun XJ , Wang MC , Zhang FH , Kong X . An integrated analysis of genome‐wide DNA methylation and gene expression data in hepatocellular carcinoma. FEBS open bio. 2018;8(7):1093‐1103. 10.1002/2211-5463.12433 PMC6026698 29988590

[42] Shen XY , Liu XP , Song CK , Wang YJ , Li S , Hu WD . Genome‐wide analysis reveals alcohol dehydrogenase 1C and secreted phosphoprotein 1 for prognostic biomarkers in lung adenocarcinoma. J Cell Physiol. 2019;234(12):22311‐22320. 31074035 10.1002/jcp.28797

[43] Ye Q‐H , Qin L‐X , Forgues M , et al. Predicting hepatitis B virus–positive metastatic hepatocellular carcinomas using gene expression profiling and supervised machine learning. Nat Med. 2003;9(4):416‐423. 12640447 10.1038/nm843

[44] Ye Q‐H , Qin L‐X , Forgues M , et al. Predicting hepatitis B virus‐positive metastatic hepatocellular carcinomas using gene expression profiling and supervised machine learning. Nat Med. 2003;9(4):416‐423. 12640447 10.1038/nm843

[45] Zhang Y , Du W , Chen Z , Xiang C . Upregulation of PD‐L1 by SPP1 mediates macrophage polarization and facilitates immune escape in lung adenocarcinoma. Exp Cell Res. 2017;359(2):449‐457. 28830685 10.1016/j.yexcr.2017.08.028

[46] Noman MZ , Desantis G , Janji B , et al. PD‐L1 is a novel direct target of HIF‐1α, and its blockade under hypoxia enhanced MDSC‐mediated T cell activation. J Exp Med. 2014;211(5):781‐790. 24778419 10.1084/jem.20131916 PMC4010891

[47] Bachmann M , Scheiermann P , Härdle L , Pfeilschifter J , Mühl H . IL‐36γ/IL‐1F9, an innate T‐bet target in myeloid cells. J Biol Chem. 2012;287(50):41684‐41696. 23095752 10.1074/jbc.M112.385443 PMC3516718

[48] Wang H , Guo J , Shang X , Wang Z . Less immune cell infiltration and worse prognosis after immunotherapy for patients with lung adenocarcinoma who harbored STK11 mutation. Int Immunopharmacol. 2020;84:106574. 32413741 10.1016/j.intimp.2020.106574

[49] Zheng C , Zheng L , Yoo J‐K , et al. Landscape of infiltrating T cells in liver cancer revealed by single‐cell sequencing. Cell. 2017;169(7):1342‐1356.e1316. 28622514 10.1016/j.cell.2017.05.035

[50] Kasper H‐U , Drebber U , Stippel D‐L , Dienes H‐P , Gillessen A . Liver tumor infiltrating lymphocytes: comparison of hepatocellular and cholangiolar carcinoma. World J Gastroenterol. 2009;15(40):5053‐5057. 19859998 10.3748/wjg.15.5053 PMC2768884

[51] Shalapour S , Lin X‐J , Bastian IN , et al. Inflammation‐induced IgA+ cells dismantle anti‐liver cancer immunity. Nature. 2017;551(7680):340‐345. 29144460 10.1038/nature24302 PMC5884449

[52] Gide TN , Quek C , Menzies AM , et al. Distinct immune cell populations define response to anti‐PD‐1 monotherapy and anti‐PD‐1/anti‐CTLA‐4 Combined therapy. Cancer Cell. 2019;35(2):238‐255.e236. 30753825 10.1016/j.ccell.2019.01.003

[53] Sangro B , Gomez‐Martin C , de la Mata M , et al. A clinical trial of CTLA‐4 blockade with tremelimumab in patients with hepatocellular carcinoma and chronic hepatitis C. J Hepatol. 2013;59(1):81‐88. 23466307 10.1016/j.jhep.2013.02.022

[54] Cai X , Gao Q , Qiu S , et al. Dendritic cell infiltration and prognosis of human hepatocellular carcinoma. J Cancer Res Clin Oncol. 2006;132(5):293‐301. 16421755 10.1007/s00432-006-0075-y PMC12161073

[55] Zhu X , Zhang J , Zhuang P , et al. High expression of macrophage colony‐stimulating factor in peritumoral liver tissue is associated with poor survival after curative resection of hepatocellular carcinoma. J Clin Oncol. 2008;26(16):2707‐2716. 18509183 10.1200/JCO.2007.15.6521

[56] Garnelo M , Tan A , Her Z , et al. Interaction between tumour‐infiltrating B cells and T cells controls the progression of hepatocellular carcinoma. Gut. 2017;66(2):342‐351. 26669617 10.1136/gutjnl-2015-310814 PMC5284473

[57] Xiao G , Deng A , Liu H , Ge G , Liu X . Activator protein 1 suppresses antitumor T‐cell function via the induction of programmed death 1. Proc Natl Acad Sci USA 2012;109(38):15419‐15424. 22949674 10.1073/pnas.1206370109 PMC3458364

[58] Mittal AK , Chaturvedi NK , Rohlfsen RA , et al. Role of CTLA4 in the proliferation and survival of chronic lymphocytic leukemia. PLoS One. 2013;8(8):e70352. 23936412 10.1371/journal.pone.0070352 PMC3731360

